# Long‐distance communication in Arabidopsis involving a self‐activating G protein

**DOI:** 10.1002/pld3.37

**Published:** 2018-02-26

**Authors:** Meral Tunc‐Ozdemir, Kang‐Ling Liao, Timothy J. Ross‐Elliott, Timothy C. Elston, Alan M. Jones

**Affiliations:** ^1^ Department of Biology University of North Carolina at Chapel Hill Chapel Hill NC USA; ^2^ Department of Pharmacology University of North Carolina at Chapel Hill Chapel Hill NC USA

**Keywords:** Arabidopsis regulator of G signaling protein 1, cotyledon, heterotrimeric G protein, photosynthesis, signaling, sugar sensing

## Abstract

In plant cells, heterotrimeric G protein signaling mediates development, biotic/abiotic stress responsiveness, hormone signaling, and extracellular sugar sensing. The amount of sugar in plant cells fluctuates from nanomolar to high millimolar concentrations over time depending on changes in the light environment. *Arabidopsis thaliana* Regulator of G Signaling protein 1 (AtRGS1) modulates G protein activation and detects the concentration and the exposure time of sugars. This is called dose–duration reciprocity in sugar sensing and occurs through AtRGS1 internalization which is directly proportional to G protein activation. One source of sugars is from CO_2_ fixation by photosynthesis. Through a simple set of experiments, we show that sugars made in cotyledons that are undergoing photomorphogenesis activate G signaling in cells distal to the nascent photosynthesis center. This occurs with sufficient speed to enable distal cells to monitor changes in photosynthetic activity in the leaves.

## 1 INTRODUCTION

In animals, the heterotrimeric G protein complex is activated by extracellular ligand binding to 7‐transmembrane G protein‐coupled receptors (GPCR). The bound GPCR catalyzes the exchange of GDP for GTP on the Gα subunit of the complex. An intrinsic GTP hydrolase activity returns the complex to its resting state (Duc, Kim, & Chung, 2017). This “off” reaction is often accelerated by a cytoplasmic Regulator of G Signaling (RGS) protein (Kehrl, 2016). GPCR internalization induced by prolonged occupancy of its cognate ligand leads to G protein inactivation and to desensitization of the signal (Rajagopal & Shenoy, 2017). Interestingly, in addition to desensitization of signal at the plasma membrane, sustained G protein signaling is achieved within the internalized compartments by β‐arrestin binding the GPCR complexes composed of a single GPCR, β‐arrestin, and G protein (Thomsen et al., 2016).

In stark contrast to animals, plants and protists have Gα subunits that spontaneously exchange guanine nucleotides, and therefore, these cells do not need or have GPCRs (Urano & Jones, 2014). Plant and protist cells have 7‐transmembrane RGS proteins that keep the complex in its inactive state (Jones, Temple, Jones, & Dohlman, 2011), and when these receptor–RGS proteins internalize through endocytosis, G signaling is self‐activated and sustained. In Arabidopsis, the Gα subunit, AtGPA1, binds either the 7‐transmembrane RGS protein, AtRGS1, or its G partner, AGB1/AGG, at the same AtGPA1 protein surface interface; therefore, we proposed that extracellular glucose shifts the equilibrium to the AtRGS1::AtGPA1 state from the AtGPA1::AGB1/AGG state (Urano, Jones et al., 2012). Consequently, the AGB1/AGG dimer is free to recruit with‐no‐lysine (WNK) kinases (Fu et al., 2014) that phosphorylate AtRGS1. Phosphorylation of AtRGS1 at Ser 428/435/436 is necessary for its internalization from the plasma membrane. The plant adaptor equivalent to β‐arrestin in animals has not yet been identified, but signaling from the endosomal compartment may be a mechanism shared with the animal G signaling pathway described earlier.

Sugar uptake and efflux between sources and sinks mediated by sugar transporters such as glucose transporters (GLUTs), sodium–glucose cotransporters (SGLTs), and SWEET proteins are important for metabolism, development, growth, and homeostasis (Han et al., 2017). The phloem transport of photosynthesis‐derived sugar into the root tip is necessary for the regulation of root elongation growth by light (Kircher & Schopfer, 2012). Glucose serves as a regulatory signal that controls expression of thousands of genes and proteins, cell‐cycle progression, metabolism, proliferation, growth, development, and stress adaptation in plants (Sheen, 2014). Perception of sugars by membrane or cytosolic sensors is also important for plant cells to adapt their activity as a function of their sugar status (Lecourieux et al., 2014). Finally, because sugars are the primary products of CO_2_ fixation, their dynamic levels in the extracellular space may inform of changes in the light environment (Liao, Jones, et al., 2017; Liao, Melvin, et al., 2017). The amount of sugar in plant cells fluctuates from nanomolar to high millimolar concentrations over time depending on changes in the light environment (Deuschle et al., 2006).

When Arabidopsis seedlings are exposed to even a small amount of light, the hypocotyls stop elongating, their superposing hooks open, the cotyledons expand, and they become photosynthetically active (Kircher & Schopfer, 2012). This so‐called de‐etiolation process is packed with many cellular changes including the groundwork for an autotrophic strategy of sugar production. Etiolated AtRGS1 null mutant seedlings have longer hypocotyls, open hooks, and expanded cotyledons (Chen, Gao, & Jones, 2006), and light‐grown mutant seedlings are hyposensitive to a high dose of glucose (Huang, Tunc‐Ozdemir, Chang, & Jones, 2015). AtRGS1 is also required for glucose‐regulated expression of certain genes (Grigston et al., 2008).

The metabolic timeline for autotrophy in Arabidopsis seedlings is not known but is probably rapid. In gymnosperms, chlorophyll synthesis and formation of the photosystems already occur in complete darkness, and illumination of cotyledons for 5 min partially activates PSII (Pavlovič, Stolárik, Nosek, Kouřil, & Ilík, 2016). In green tissue, sucrose accumulation begins within 15 min of the onset of illumination, and Glc‐1‐P and Glc‐6‐P increase rapidly in response to illumination to reach saturation within 5 min (Okumura, Inoue, Kuwata, & Kinoshita, 2016). Genes involved in photosynthesis and its regulation dominate transcripts specific to the greening cotyledon (Li, Swaminathan, & Hudson, 2011).

Besides the signaling role for extracellular sugar in plant development and cell behavior, the presence of postphloem transport of sucrose to recipient sink cells that may occur either apoplastically into cell wall matrix or symplastically through plasmodesmata is well known (Rolland, Baena‐Gonzalez, & Sheen, 2006; Wang & Ruan, 2013). Extracellular glucose is acting as a signal, but it is not clear specifically what behavior it is provoking. Nonetheless, speculation abounds. The hydrolysis of sucrose by cell wall invertases into glucose and fructose is essential for appropriate metabolism, growth, and differentiation in plants (Sherson, Alford, Forbes, Wallace, & Smith, 2003). Extracellular invertases are also required for plants' abilities to regrow and ultimately compensate for fitness following apical damage (Siddappaji et al., 2015). A signaling role for glucose in development has been accepted for decades. For example, the classic experiments from the 1960s such as performed by the late Ralph Wetmore and Donald Northcote showed unequivocally that extracellular sugar is a morphogenic signal in cell differentiation (Jeffs & Northcote, 1967; Wetmore & Rier, 1963). Sugar controls the plant cell cycle (Riou‐Khamlichi, Menges, Healy, & Murray, 2000), and it is essential for the activation of cell division at the shoot apex in auxin‐dependent manner (Li et al., 2017; Raya‐González et al., 2017). In addition, the concept designated by Sweet Immunity presumes that extracellular sugars are “danger signals” in immune and defense responses (Bolouri Moghaddam & Van den Ende, 2013).

While AtRGS1 is the strongest candidate for an extracellular glucose sensor based on genetic evidence, to date, there is no direct evidence that AtRGS1 binds glucose. However, the AtRGS1 topology analogous to GPCRs (Chen et al., 2003) and the exogenous glucose‐induced AtRGS1 endocytosis (Fu et al., 2014; Urano, Phan et al., 2012) are consistent with extracellular glucose perception.

Because heterotrimeric G proteins are involved in the many cellular functions mentioned earlier including immunity, morphogenesis, abiotic stress responses, and growth (Colaneri, Tunc‐Ozdemir, Huang, & Jones, 2014; Tunc‐Ozdemir & Jones, 2017a; Ueguchi‐Tanaka et al., 2000; Ullah et al., 2001; Urano et al., 2016) and AtRGS1 internalizes in response to sugars, we speculated that the AtRGS1/G protein complex monitors sugar levels produced by photosynthesis. Given that AtRGS1 null mutants are unable to control photosynthesis efficiency in a dynamic light environment (Liao, Jones, et al., 2017) and are hyposensitive to high glucose, AtRGS1 may be the extracellular glucose sensor in distal cells. It is also consistent with previous studies showing that the G protein complex is required for a high light response (Galvez‐Valdivieso et al., 2009).

Given that hypocotyl elongation, a sugar‐mediated pathway, is partially mediated by BRL3 and AtRGS1 (Tunc‐Ozdemir & Jones, 2017b) specifically in dark, *rgs1* mutants had longer hypocotyl (Chen et al., 2006), and null alleles of *AtRGS1* confer increased cell division in the root apical meristem (Chen et al., 2003), we propose that AtRGS1 in epidermal hypocotyl cells evaluates photosynthetic activity of the distal greening cotyledon cells with a time constant of minutes. Light irradiated on the cotyledons activates G signaling in the hypocotyl. This requires photosynthetically active wavelengths of light and CO_2_ and is sensitive to photosynthesis inhibitors. This light‐induced signal from the cotyledons can be replaced by D‐glucose but not L‐glucose applied at the distal cotyledon site.

## 2 METHOD

### 2.1 Plant growth

Arabidopsis expressing *AtRGS1‐YFP* (encoding 1‐459aa) (Col‐0) in pEarleyGate101, containing a 35S promoter and C‐terminal YFP‐HA (ABRC Stock: CS69139) (Huang et al., 2015) was sterilized with ethanol (70% for 10 min then 95% for 10 min) and stratified on plates containing ¼ Murashige and Skoog (MS) liquid media including 0.025% MES at 5°C for 2 days. This was followed by 2 hr 100 μmol m^−2 ^s^−1^ light and then grown in near darkness (<1 μmol m^−2 ^s^−1^) at 27°C for 3 days on a shaker.

### 2.2 Light irradiation

For white light illumination of whole seedling, the seedlings were illuminated with 480 μmol m^−2 ^s^−1^ light in sterile water. For partial illumination experiments, either the tissue was covered with aluminum foil, or the cotyledons were excised before light exposure. In one experiment, seedlings were illuminated either with photosynthetically active, red (600–700 nm), or nonactive green (400–500 nm) light at an intensity of 480 μE m^−2^ s^−1^ for 30 and 60 min. Green and red monochromatic lights were obtained by passing the white light from the halogen projector lamp through B5‐5400 or B5‐6800 interference filters, respectively (Baird Atomic, Inc.).

### 2.3 Inhibiting photosynthesis

For PSII inhibition, 100 μM DCMU was dissolved in 0.01% ethanol (EtOH). First seedlings were stratified at 5°C for 2 days and then grown in ~40 μmol m^−2 ^s^−1^ light at 27°C for 2 days on a shaker. Next, the seedlings were treated either with 100 μM DCMU (Sigma) or 0.01% EtOH alone (control case) under ~40 μmol m^−2 ^s^−1^ light exposure on a shaker for 1 hr, and then, plates including seedlings were covered with foil and were grown at 27°C for another 1 day on a shaker before the light illumination experiments were performed. Finally, each seedling was placed on a slide with either 0.01% EtOH or 100 μM DCMU with ¼ MS, and then, the whole seedlings were either kept in 60 min in dark or exposed to ~480 μmol m^−2 ^s^−1^ light for 60 min. For PSI inhibition, 0 or 30 μM paraquat dissolved in water was added to whole seedlings kept in 60 min in darkness or under ~480 μmol m^−2 ^s^−1^ light. To test the light‐induced AtRGS1 endocytosis in the absence of CO_2,_ the seedlings were immersed in degassed water by submerging them in sealed glass slide sandwiches. CO_2_ is soluble in water, but its diffusion rate is 10,000 slower than in air (Mommer & Visser, 2005). To demonstrate glucose‐induced G activation, cotyledons were removed and replaced with ¼ MS solid media (with 0.5% phytoagar) including 0 or 6% sucrose, mannitol, or D‐/L‐glucose.

### 2.4 Imaging

Hypocotyl epidermal cells located 2–5 mm below the cotyledons were imaged using vertical optical sectioning (z‐stack acquisition). A Zeiss LSM710 confocal laser scanning microscope with C‐Apochromat × 40/1.2 water immersion objective was used to focus a diode laser (489 nm excitation) to excite YFP. Emission was detected between 526 nm and 569 nm with a photomultiplier detector. ImageJ software was used to analyze fluorescence internalization representing G protein activation. The experiments were repeated three times using independent biological replicates with similar results, and one representative data set with error bars showing the ± standard error of the mean (*SEM*) is depicted.

### 2.5 Transmission electron microscopy

Arabidopsis cotyledons and hypocotyls grown ¼ MS liquid media in aluminum foil‐covered plates were illuminated with ~480 μmol m^−2 ^s^−1^ light for 10 min and immersion‐fixed in 4% paraformaldehyde/3% glutaraldehyde/0.05 M sodium phosphate, pH 7.4, overnight at 4°C. After three buffer washes (w/0.05 M NaPhos), the samples were postfixed in 1% osmium tetroxide/0.05 M sodium phosphate, pH 7.4, for 1 hr followed by three washes in deionized water. Cotyledons and hypocotyls were stained *en bloc* with 2% aqueous uranyl acetate, dehydrated through a graded series of ethanol (30%, 50%, 75%, 90%, 100% X3) and propylene oxide, infiltrated, and embedded in Spurr's epoxy resin (Polysciences, Warrington, PA). Using a diamond knife, 1‐μm longitudinal and transverse sections were cut, mounted on slides, and stained with 1% toluidine blue and examined by light microscopy to isolate the region of interest. Ultrathin sections (70–80 nm) were cut with a diamond knife and mounted on 200 mesh copper grids followed by staining with 4% aqueous uranyl acetate for 12 min and lead citrate for 8 min. Grids were observed using a JEOL JEM‐1230 transmission electron microscope operating at 80 kV (JEOL USA, Inc., Peabody, MA), and images were acquired with a Gatan Orius SC1000 CCD Digital Camera and Gatan Microscopy Suite 3.0 software (Gatan, Inc., Pleasanton, CA).

## 3 RESULTS AND DISCUSSION

**Figure 1:**
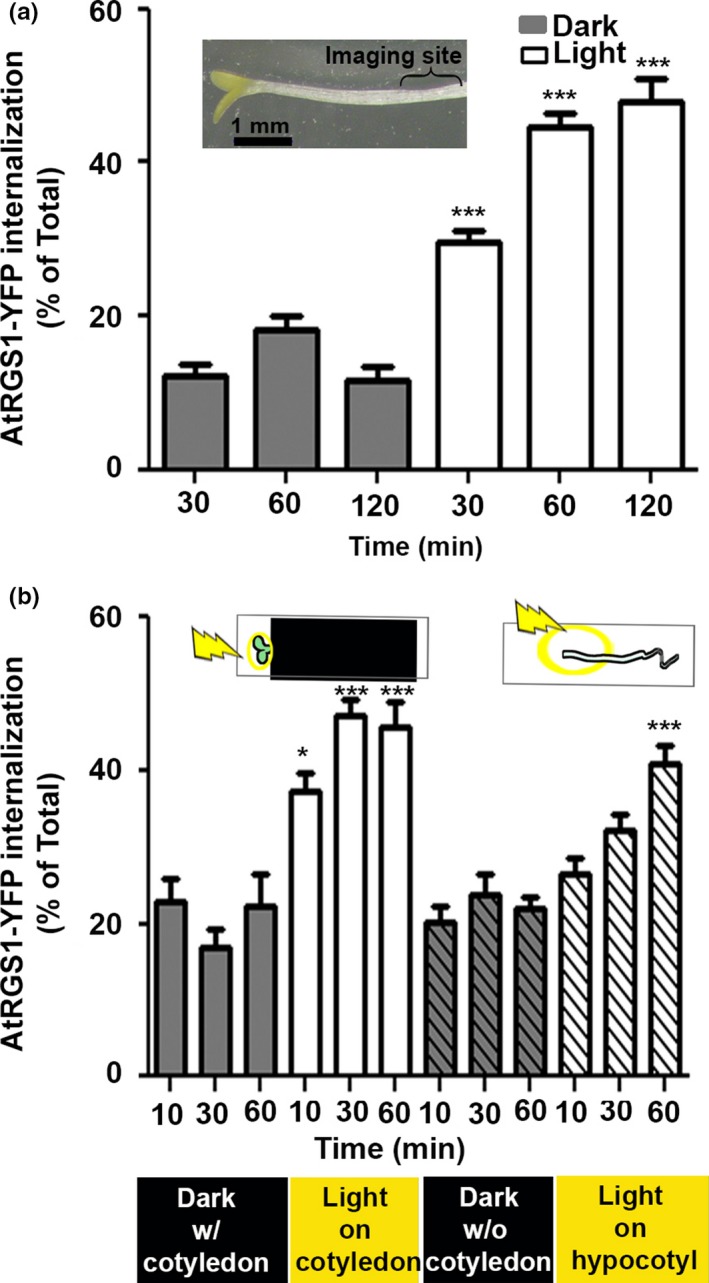
Light induces G protein signal activation. (a) Light illumination of whole seedling activates G signaling as proxied by the proportion of AtRGS1 endocytosis in hypocotyls as described in Fu et al. (2014). The inset shows partially de‐etiolated seedling used for this experiment. White bars represent measured AtRGS1‐YFP internalization over time in the hypocotyl regions shown with the bracket on inset located 2–5 mm below the cotyledons. (*n* = 3–28). (b) A signal originating in the cotyledon is required for G activation in hypocotyls. Light was illuminated only on the cotyledon (solid white bars) or on hypocotyls of seedlings without cotyledons (white hatched‐filled bars). AtRGS1‐YFP internalization was quantitated over time in distal cells in the hypocotyl regions shown in c. (*n* = 3–28). a–b, Error bars show standard error of the mean (*SEM*); *p <* *.05, ***p <* *.001 (two‐tailed *t* test)

Approximately half of the fixed CO_2_ is immediately converted to starch while the rest is mobilized (Mengin et al., 2017; Sharkey, Berry, & Raschke, 1985; Sulpice et al., 2014). Given the rapid speed that fixed carbon reaches distal cells (Mudgil et al., 2016), we hypothesized that the extracellular sugars that are detected by AtRGS1 are derived by CO_2_ fixation. As such, detection of G signaling activation in response to illumination is expected. To test this, we used the standard assay for G protein activation, namely AtRGS1 endocytosis (Fu et al., 2014). The proportion of the total pool of AtRGS1 that is internalized is directly proportional to the amount of G protein activation (Figure S5 of Fu et al., 2014). The seedlings used in the initial part of this study were partially de‐etiolated having elongated hypocotyls, opened hooks, and yellow‐green cotyledons (Figure 1a, inset). Hypocotyl epidermal cells located 2–5 mm below the cotyledons (Figure 1a, [inset] bracket) were imaged using vertical optical sectioning (z‐stack acquisition).

**Figure 2:**
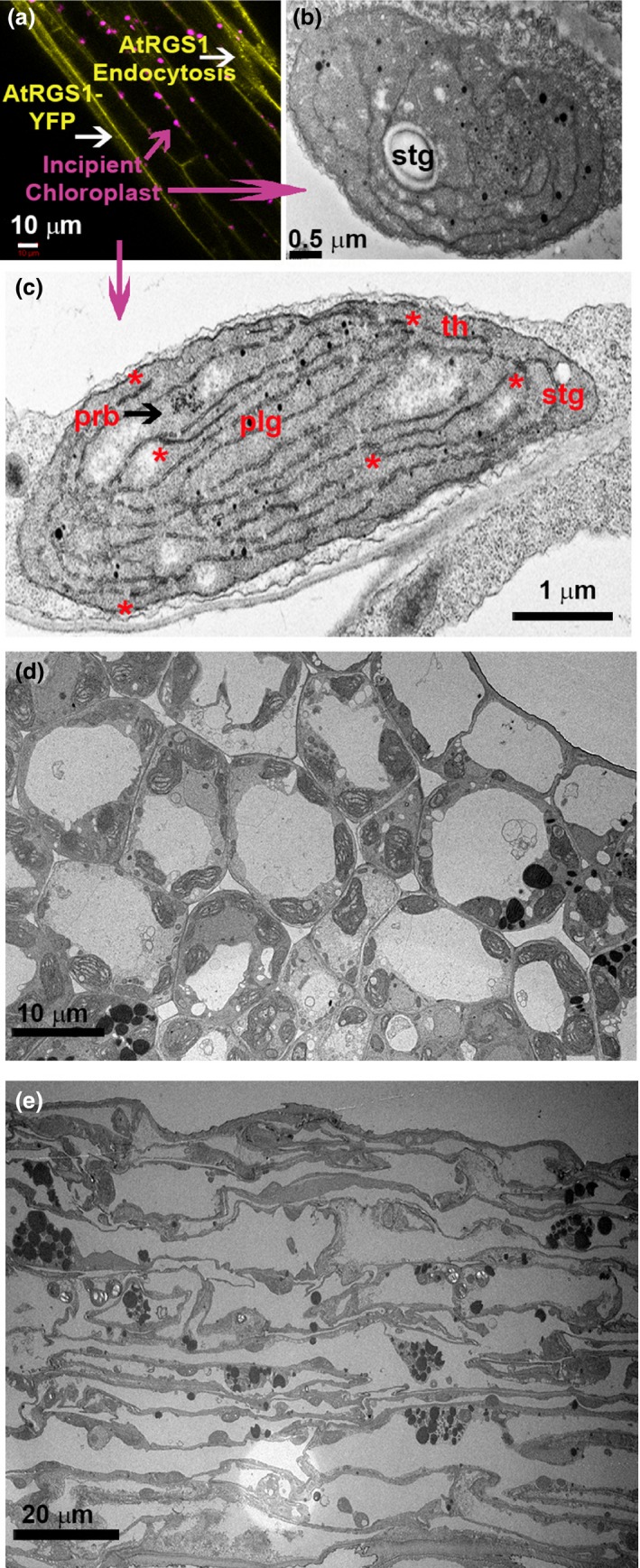
Transmission electron microscopy of cotyledon and hypocotyl epidermal cells shows early chloroplasts. (a) AtRGS1‐YFP internalization is yellow vesicles, and early chloroplasts in hypocotyl epidermal cell are colored in magenta and imaged with transmission electron microscopy. (b) In hypocotyl epidermal cells and (c) cotyledon illuminated for 10‐min remnants of the prolamellar body (prb), which is membrane aggregation of semicrystalline lattices of branched tubules (black arrowhead), developing thylakoids (th) with some incipient grana stacks (asterisks), starch granules (stg), and plastoglobules (plg) are shown. (d and e) Lower magnification of cotyledon and hypocotyl sections illuminated for 10 min

Within 30 min of light irradiation (480 μmol m^−2 ^s^−1^) of whole seedlings, activation of G signaling was detected in hypocotyl epidermal cells located 2–5 mm below the cotyledon (*p *<* *.0001; Figure 1a). Activation was fast with near‐maximal output within 10 min. Next, we isolated the primary photoperception tissue to the greening cotyledons by covering the hypocotyl and root and illuminating the area of the cotyledon (480 μmol m^−2 ^s^−1^). Like what was observed with whole seedling illumination, there was an increase in G protein activation (*p *<* *.0001) (Figure 1b, solid white bars) in hypocotyl cells 2–5 mm distill to the cotyledons. To test a photoperception role for the chloroplasts, cotyledons were removed leaving only the relatively few early chloroplasts distributed along the hypocotyl upon light illumination (Figure 2a,b). Transmission electron microscopy of cotyledon and hypocotyl epidermal cells illuminated for 10 min revealed some etioplasts but mostly early chloroplasts. As shown in Figure 2c, the evidence supporting this was as follows: The prolamellar body (prb) (i) lost its crystalline array (black arrowhead), (ii) developed thylakoids (th) (iii) displayed incipient grana stacks (asterisks), and (iv) displayed starch granules (stg) and plastoglobules (plg).

Removal of the cotyledons did not change the basal level activation (Figure 1b, gray hatched‐filled bars) but greatly reduced the kinetics and amplitude of light‐induced G protein activation (Figure 1b, white hatched‐filled bars). This indicates that G activation reported by AtRGS1 internalization is not a general stress response. It also shows that cotyledons are the main source of the long‐distance signal that activates G signaling.

**Figure 3:**
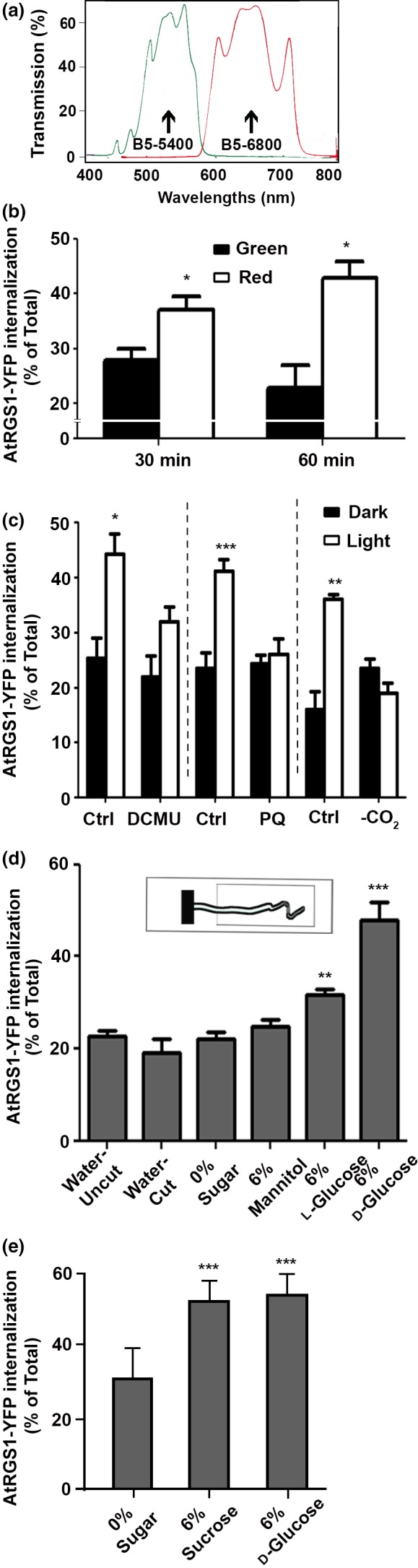
Photosynthesis‐dependent sugars are the light‐induced signal for G protein activation. (a) Spectra of B5‐6800 or 5400 interference filters (Baird Atomic, Inc.) used for irradiation. (b) A photosynthesis product is the signal triggering G protein activation in hypocotyls. Photosynthetically active red light illumination by passing the white light from the halogen projector lamp through a B5‐6800 interference filter for 30 min or 60 min induced AtRGS1 endocytosis while inactive green light did not. (c) PSII and PSI are required for G protein activation. Seedlings pretreated either with 0.01% EtOH (Control, Ctrl), the PSII inhibitor, DCMU, or the PSI inhibitor (paraquat, PQ) were either kept in dark or exposed to light. CO_2_ was reduced (‐CO_2_) by submerging seedlings in degassed water before and during irradiation. (d) Photosynthesis‐produced sugar is the cue for G protein activation. Cotyledons were removed and replaced with ¼ MS solid media block including 0.5% phytoagar, 0 or 6% D‐/L‐glucose or mannitol as shown in the graphic inset. (e) Photosynthesis‐produced sucrose/glucose is the cue for G protein activation. Cotyledons were removed and replaced with ¼ MS solid media block including 0.5% phytoagar and 0 or 6% D‐glucose or sucrose as shown in the graphic inset. b‐e, The experiments were repeated three times using independent biological replicates with similar results, and one representative data set with error bars showing the ±*SEM* (*n* = 3–11) is depicted. *p <* *.05 **p <* *.01, ***p <* *.001 (two‐tailed *t* test)

To determine whether the cotyledon‐derived signal that distally activates G signaling requires the light reactions of photosynthesis, seedlings were irradiated with photosynthetically active (600–700 nm; red) and nonactive (400–500 nm; green) (Figure 3a) light. G protein activation was significantly higher under red light compared to the same intensity green light both at 30‐ and 60‐min irradiation (480 μmol m^−2 ^s^−1^, *p *<* *.05, Figure 3b). This is consistent with G protein activation as a photosynthesis‐dependent process.

To verify a role for photosynthesis, seedlings were treated with 3‐(3,4‐dichlorophenyl)‐1,1‐dimethylurea (DCMU), a herbicide that reduces the quantum yield of PSII (Galatro, Puntarulo, Guiamet, & Simontacchi, 2013). Seedlings were treated either with 100 μM DCMU (Sigma) or 0.01% EtOH alone (control case), and then, the seedlings were kept in dark or exposed to 480 μmol m^−2 ^s^−1^ light for 60 min. DCMU impaired the light‐induced AtRGS1 endocytosis that increases at 60 min after treatment (Figure 3c) and had no effect on basal AtRGS1 endocytosis without light.

To determine whether PSI is required for light‐induced G protein activation, we tested the effect of paraquat. Paraquat is a well‐characterized electron acceptor that inhibits PSI, specifically oxidizing ferredoxin (Fan, Jia, Barber, & Chow, 2009). Seedlings in 0 or 30 μM paraquat were kept in darkness or irradiated with 480 μmol m^−2 ^s^−1^ light for 60 min, and activation was measured (Figure 3c). While there was a ~ 2‐fold increase in activation (*p *<* *.0001) in the 0 μM paraquat seedlings exposed to light, light‐induced activation was not detected in the presence of 30 μM paraquat.

To determine whether light‐induced activation required CO_2_ fixation, seedlings were immersed in the medium prior to irradiation. Submergence attenuates gas exchange between the plant and the environment by 10^4^‐fold due to the lower diffusion rate of gases in water versus air (Mommer & Visser, 2005). G protein activation was greatly reduced when CO_2_ was limited (Figure 3d). Taken together, the requirements for PSII, PSI, and CO_2_ support the notion that a cotyledon‐derived photosynthesis product is the signal in G protein activation in cells distill to the leaf. We do not exclude that multiple signals are involved.

Fixed carbon in the leaf is rapidly distributed as sugars to sink tissue in plants with rates of movement ~1 cm per minute (Mudgil et al., 2016); thus, sugars are candidates for the long‐distance activator. To determine whether a sugar is the photosynthesis‐dependent product that activates G signaling, cotyledons were excised and replaced with a ¼x MS agar cube containing 6% D‐glucose. After 90 min, distal application of glucose increased AtRGS1 internalization (*p *<* *.0001) compared to the 0% glucose control (Figure 3d). A similar response was not seen with the osmotic control, 6% mannitol. The observation that 6% L‐glucose had little effect indicates stereospecificity in the glucose response. Because sucrose is a long‐distance signal that controls root growth (Kircher & Schopfer, 2012), we also looked at the effect of distal sucrose application in AtRGS1 endocytosis (Figure 3e) and found that 6% sucrose has similar effect as glucose and increases AtRGS1 endocytosis (*p *<* *.0001).

**Figure 4:**
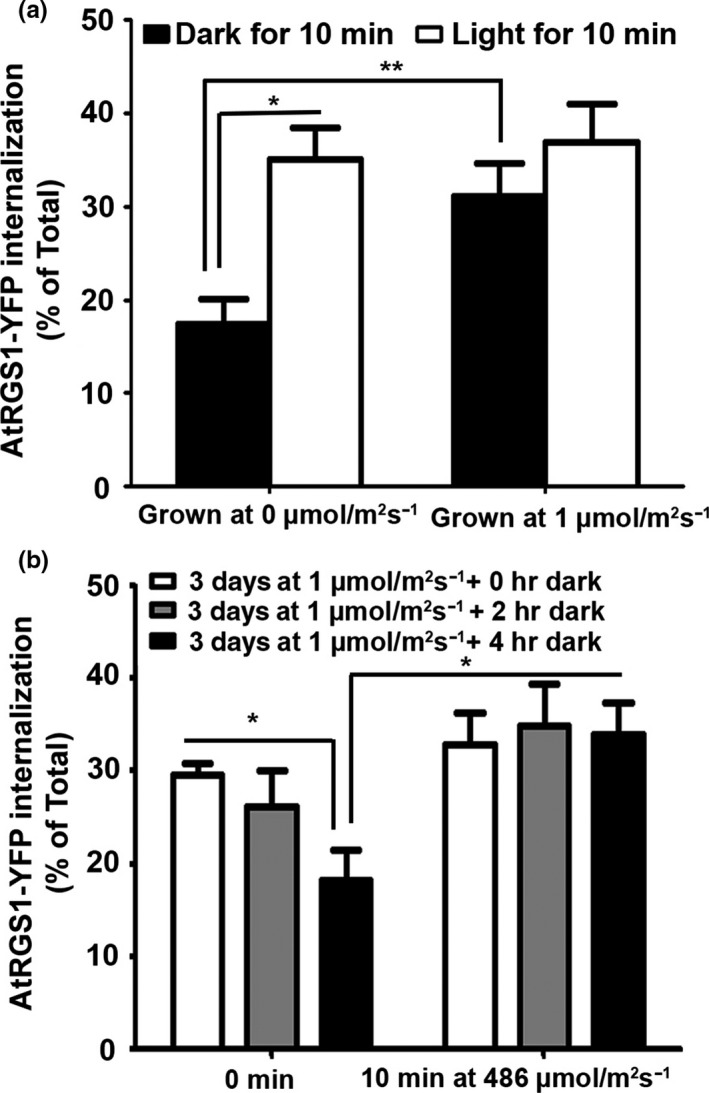
Light activation of G signaling in long hypocotyl green seedlings requires dark adaptation. (a) Hypocotyls grown in 1 μmol m^−2 ^s^−1^ light and hypocotyls grown in near darkness (<0.04 μmol m^−2 ^s^−1^, depicted as 0 μmol m^−2 ^s^−1^) at 27°C for 3 days on a shaker were illuminated with 486 μmol m^−2 ^s^−1^ light for minute, and AtRGS1 endocytosis is measured. (b) Hypocotyls grown in 1 μmol m^−2 ^s^−1^ light were dark‐adapted at 27°C for 2 and 4 hr, and then, they were illuminated with 486 μmol m^−2 ^s^−1^ light for 10 min. The experiments were repeated three times using independent biological replicates with similar results, and one representative data set with error bars showing the ±*SEM* (*n* = 3–6) is depicted. *p < .05 **p < .01, (two‐tailed *t* test)

Glucose sensing by the AtRGS1/G protein complex utilizes what we have termed dose–duration reciprocity (Fu et al., 2014), where both time and amount of glucose are used to affect the signal‐induced outcome. This is a complex mechanism that employs regulatory loops to achieve adaptive behavior, filters, and memory (Liao, Jones, et al., 2017). The rate of glucose produced is a function of the level of irradiance, photosynthetic efficiency, and carbon fixation capacity (e.g., the number of mature chloroplasts). Naturally, for the same amount of low irradiance, fully green seedlings will produce more glucose than partially etiolated seedlings. As expected, we found that in hypocotyl epidermal cells of seedlings that had fully green cotyledons, AtRGS1 endocytosis was already maximum (specifically ~ 2‐fold higher than the tissue kept in near darkness (*p *<* *.0001)). Light illumination of the seedlings for 10 min resulted in no increase in AtRGS1 internalization (Figure 4a). However, if they were dark‐adapted until they returned to baseline for 4 hr, then AtRGS1 endocytosis is activated again in 10 min (Figure 4b). Therefore, the question arises to why plants have an extracellular glucose sensor that is so sensitive that it is already at maximum activation under low light conditions. We speculate on two possible reasons: (i) The AtRGS1/G complex only operates under low light and during photomorphogenesis when full photosynthetic capacity has not yet been reached such as with the seedlings used in this study. However, this is inconsistent with the genetic data showing clear phenotypes for light‐grown *rgs1* mutants suggesting that mature plants need the AtRGS1/G complex. (ii) More likely is that the dynamic range for extracellular glucose detection by AtRGS1/G complex is tunable, being the most sensitive when extracellular concentrations are stably low (e.g., partially de‐etiolated or plants at dawn or after a prolonged shadow) and less sensitive when stably high (e.g., plants in full sun at noon). Supporting this idea of tunability of the dynamic range for extracellular glucose detection is the finding that seedling sensitivity to extracellular glucose is a nonlinear function of the AtRGS1 pool size (Liao, Melvin, et al., 2017) which is itself under glucose control (Yan, Wang, Fu, & Chen, 2017). This is also consistent with an AtRGS1 role as a shadow detector which senses large changes in light while at the same time filters types of fluctuation in light that do not affect photosynthesis efficiency (Liao, Jones, et al., 2017).

The results here also clearly dispel the idea that AtRGS1 internalization is simply a reaction to stress because many of the treatments performed here are stressful (surgery, chemical inhibitors, acapnia), yet we showed that they blocked, rather than promoted, light‐induced activation of G signaling and had no other effect on the baseline.

We showed with a simple set of experiments that information on the changes in the light environment affecting sugar production is conveyed to hypocotyls through photosynthesis‐generated glucose. This work establishes a new basis by which cells distal to the leaf can monitor photosynthesis product(s) and provides a paradigm for plant sink tissues detecting light changes at source tissues.

## References

[pld337-bib-0001] Bolouri Moghaddam, M. R. , & Van den Ende, W. (2013). Sweet immunity in the plant circadian regulatory network. Journal of Experimental Botany, 64, 1439–1449. 10.1093/jxb/ert046 23564957

[pld337-bib-0002] Chen, J.‐G. , Gao, Y. , & Jones, A. M. (2006). Differential roles of Arabidopsis heterotrimeric G‐protein subunits in modulating cell division in roots. Plant Physiology, 141, 887–897. 10.1104/pp.106.079202 16679415 PMC1489905

[pld337-bib-0003] Chen, J.‐G. , Willard, F. S. , Huang, J. , Liang, J. , Chasse, S. A. , Jones, A. M. , & Siderovski, D. P. (2003). A seven‐transmembrane RGS protein that modulates plant cell proliferation. Science, 301, 1728–1731. 10.1126/science.1087790 14500984

[pld337-bib-0004] Colaneri, A. C. , Tunc‐Ozdemir, M. , Huang, J. P. , & Jones, A. M. (2014). Growth attenuation under saline stress is mediated by the heterotrimeric G protein complex. BMC Plant Biology, 14, 129 10.1186/1471-2229-14-129 24884438 PMC4061919

[pld337-bib-0005] Deuschle, K. , Chaudhuri, B. , Okumoto, S. , Lager, I. , Lalonde, S. , & Frommer, W. B. (2006). Rapid metabolism of glucose detected with FRET glucose nanosensors in epidermal cells and intact roots of arabidopsis RNA‐silencing mutants. Plant Cell Online, 18, 2314–2325. 10.1105/tpc.106.044073 PMC1560921 16935985

[pld337-bib-0006] Duc, N. M. , Kim, H. R. , & Chung, K. Y. (2017). Recent progress in understanding the conformational mechanism of heterotrimeric G protein activation. Biomolecules & Therapeutics, 25, 4–11. 10.4062/biomolther.2016.169 28035078 PMC5207459

[pld337-bib-0007] Fan, D.‐Y. , Jia, H. , Barber, J. , & Chow, W. S. (2009). Novel effects of methyl viologen on photosystem II function in spinach leaves. European Biophysics Journal, 39, 191–199. 10.1007/s00249-009-0484-3 19495738

[pld337-bib-0008] Fu, Y. , Lim, S. , Urano, D. , Tunc‐Ozdemir, M. , Phan, N. G. , Elston, T. C. , & Jones, A. M. (2014). Reciprocal encoding of signal intensity and duration in a glucose‐sensing circuit. Cell, 156, 1084–1095. 10.1016/j.cell.2014.01.013 24581502 PMC4364031

[pld337-bib-0009] Galatro, A. , Puntarulo, S. , Guiamet, J. J. , & Simontacchi, M. (2013). Chloroplast functionality has a positive effect on nitric oxide level in soybean cotyledons. Plant Physiology and Biochemistry, 66, 26–33. 10.1016/j.plaphy.2013.01.019 23466744

[pld337-bib-0010] Galvez‐Valdivieso, G. , Fryer, M. J. , Lawson, T. , Slattery, K. , Truman, W. , Smirnoff, N. , … Mullineaux, P. M. (2009). The high light response in Arabidopsis involves ABA signaling between vascular and bundle sheath cells. Plant Cell, 21, 2143–2162. 10.1105/tpc.108.061507 19638476 PMC2729609

[pld337-bib-0011] Grigston, J. C. , Osuna, D. , Scheible, W.‐R. , Liu, C. , Stitt, M. , & Jones, A. M. (2008). D‐Glucose sensing by a plasma membrane regulator of G signaling protein, AtRGS1. FEBS Letters, 582, 3577–3584. 10.1016/j.febslet.2008.08.038 18817773 PMC2764299

[pld337-bib-0012] Han, L. , Zhu, Y. , Liu, M. , Zhou, Y. , Lu, G. , Lan, L. , … Zhang, X. C. (2017). Molecular mechanism of substrate recognition and transport by the AtSWEET13 sugar transporter. Proceedings of the National Academy of Sciences, 114, 10089–10094. 10.1073/pnas.1709241114 PMC5617298 28878024

[pld337-bib-0013] Huang, J.‐P. , Tunc‐Ozdemir, M. , Chang, Y. , & Jones, A. M. (2015). Cooperative control between AtRGS1 and AtHXK1 in a WD40‐repeat protein pathway in Arabidopsis thaliana. Frontiers in Plant Science, 6, 851. 26528314 10.3389/fpls.2015.00851 PMC4602111

[pld337-bib-0014] Jeffs, R. A. , & Northcote, D. H. (1967). The influence of indol‐3yl acetic acid and sugar on the pattern of induced differentiation in plant tissue culture. Journal of Cell Science, 2, 77–88. 6031009 10.1242/jcs.2.1.77

[pld337-bib-0015] Jones, J. C. , Temple, B. R. S. , Jones, A. M. , & Dohlman, H. G. (2011). Functional Reconstitution of an Atypical G Protein Heterotrimer and Regulator of G Protein Signaling Protein (RGS1) from *Arabidopsis thaliana* . Journal of Biological Chemistry, 286, 13143–13150. 10.1074/jbc.M110.190355 21325279 PMC3075661

[pld337-bib-0016] Kehrl, J. H. (2016). The impact of RGS and other G‐protein regulatory proteins on Gαi‐mediated signaling in immunity. Biochemical Pharmacology, 114, 40–52. 10.1016/j.bcp.2016.04.005 27071343 PMC4993105

[pld337-bib-0017] Kircher, S. , & Schopfer, P. (2012). Photosynthetic sucrose acts as cotyledon‐derived long‐distance signal to control root growth during early seedling development in Arabidopsis. Proceedings of the National Academy of Sciences USA, 109, 11217–11221. 10.1073/pnas.1203746109 PMC3396492 22733756

[pld337-bib-0018] Lecourieux, F. , Kappel, C. , Lecourieux, D. , Serrano, A. , Torres, E. , Arce‐Johnson, P. , & Delrot, S. (2014). An update on sugar transport and signalling in grapevine. Journal of Experimental Botany, 65, 821–832. 10.1093/jxb/ert394 24323501

[pld337-bib-0019] Li, X. , Cai, W. , Liu, Y. , Li, H. , Fu, L. , Liu, Z. , … Xiong, Y. (2017). Differential TOR activation and cell proliferation in *Arabidopsis* root and shoot apexes. Proceedings of the National Academy of Sciences, 114, 2765–2770. 10.1073/pnas.1618782114 PMC5347562 28223530

[pld337-bib-0020] Li, Y. , Swaminathan, K. , & Hudson, M. E. (2011). Rapid, organ‐specific transcriptional responses to light regulate photomorphogenic development in dicot seedlings. Plant Physiology, 156, 2124–2140. 10.1104/pp.111.179416 21653191 PMC3149948

[pld337-bib-0021] Liao, K.‐L. , Jones, R. D. , McCarter, P. , Tunc‐Ozdemir, M. , Draper, J. A. , Elston, T. C. , … Jones, A. M. (2017). A shadow detector for photosynthesis efficiency. Journal of Theoretical Biology, 414, 231–244. 27923735 10.1016/j.jtbi.2016.11.027 PMC5635846

[pld337-bib-0022] Liao, K.‐L. , Melvin, C. E. , Sozzani, R. , Jones, R. D. , Elston, T. C. , & Jones, A. M. (2017). Dose‐Duration Reciprocity for G protein activation: Modulation of kinase to substrate ratio alters cell signaling. PLoS ONE, 12, e0190000. 29287086 10.1371/journal.pone.0190000 PMC5747438

[pld337-bib-0023] Mengin, V. , Pyl, E.‐T. , Alexandre Moraes, T. , Sulpice, R. , Krohn, N. , Encke, B. , & Stitt, M. (2017). Photosynthate partitioning to starch in Arabidopsis thaliana is insensitive to light intensity but sensitive to photoperiod due to a restriction on growth in the light in short photoperiods. Plant, Cell & Environment, 40, 2608–2627. 10.1111/pce.13000 28628949

[pld337-bib-0024] Mommer, L. , & Visser, E. J. W. (2005). Underwater Photosynthesis in Flooded Terrestrial Plants: A Matter of Leaf Plasticity. Annals of Botany, 96, 581–589. 10.1093/aob/mci212 16024559 PMC4247027

[pld337-bib-0025] Mudgil, Y. , Karve, A. , Teixeira, P. J. P. L. , Jiang, K. , Tunc‐Ozdemir, M. , & Jones, A. M. (2016). Photosynthate regulation of the root system architecture mediated by the heterotrimeric G protein complex in arabidopsis. Frontiers in Plant Science, 7, 1255. 27610112 10.3389/fpls.2016.01255 PMC4997095

[pld337-bib-0026] Okumura, M. , Inoue, S.‐I. , Kuwata, K. , & Kinoshita, T. (2016). Photosynthesis activates plasma membrane H + ‐ATPase via sugar accumulation. Plant Physiology, 171, 580–589. 10.1104/pp.16.00355 27016447 PMC4854722

[pld337-bib-0027] Pavlovič, A. , Stolárik, T. , Nosek, L. , Kouřil, R. , & Ilík, P. (2016). Light‐induced gradual activation of photosystem II in dark‐grown Norway spruce seedlings. Biochimica et Biophysica Acta, 1857, 799–809. 10.1016/j.bbabio.2016.02.009 26901522

[pld337-bib-0028] Rajagopal, S. , & Shenoy, S. K. (2017). GPCR desensitization: Acute and prolonged phases. Cellular Signalling, 41, 9–16. 28137506 10.1016/j.cellsig.2017.01.024 PMC5533627

[pld337-bib-0029] Raya‐González, J. , López‐Bucio, J. S. , Prado‐Rodríguez, J. C. , Ruiz‐Herrera, L. F. , Guevara‐García, Á. A. , & López‐Bucio, J. (2017). The MEDIATOR genes MED12 and MED13 control Arabidopsis root system configuration influencing sugar and auxin responses. Plant Molecular Biology, 95, 141–156. 10.1007/s11103-017-0647-z 28780645

[pld337-bib-0030] Riou‐Khamlichi, C. , Menges, M. , Healy, J. M. , & Murray, J. A. (2000). Sugar control of the plant cell cycle: Differential regulation of Arabidopsis D‐type cyclin gene expression. Molecular and Cellular Biology, 20, 4513–4521. 10.1128/MCB.20.13.4513-4521.2000 10848578 PMC85832

[pld337-bib-0031] Rolland, F. , Baena‐Gonzalez, E. , & Sheen, J. (2006). Sugar sensing and signaling in plants: Conserved and novel mechanisms. Annual Review of Plant Biology, 57, 675–709. 10.1146/annurev.arplant.57.032905.105441 16669778

[pld337-bib-0032] Sharkey, T. D. , Berry, J. A. , & Raschke, K. (1985). Starch and sucrose synthesis in phaseolus vulgaris as affected by light, CO(2), and abscisic acid. Plant Physiology, 77, 617–620. 10.1104/pp.77.3.617 16664108 PMC1064574

[pld337-bib-0033] Sheen, J. (2014). Master regulators in plant glucose signaling networks. Journal of Plant Biology, 57, 67–79. 10.1007/s12374-014-0902-7 25530701 PMC4270195

[pld337-bib-0034] Sherson, S. M. , Alford, H. L. , Forbes, S. M. , Wallace, G. , & Smith, S. M. (2003). Roles of cell‐wall invertases and monosaccharide transporters in the growth and development of Arabidopsis. Journal of Experimental Botany, 54, 525–531. 10.1093/jxb/erg055 12508063

[pld337-bib-0035] Siddappaji, M. H. , Scholes, D. R. , Krishnankutty, S. M. , Calla, B. , Clough, S. J. , Zielinski, R. E. , & Paige, K. N. (2015). The role of invertases in plant compensatory responses to simulated herbivory. BMC Plant Biology, 15, 278 10.1186/s12870-015-0655-6 26572986 PMC4647499

[pld337-bib-0036] Sulpice, R. , Flis, A. , Ivakov, A. A. , Apelt, F. , Krohn, N. , Encke, B. , … Stitt, M. (2014). Arabidopsis coordinates the diurnal regulation of carbon allocation and growth across a wide range of photoperiods. Molecular Plant, 7, 137–155. 10.1093/mp/sst127 24121291

[pld337-bib-0037] Thomsen, A. R. B. , Plouffe, B. , Cahill, T. J. , Shukla, A. K. , Tarrasch, J. T. , Dosey, A. M. , … Lefkowitz, R. J. (2016). GPCR‐G protein‐β‐arrestin super‐complex mediates sustained G protein signaling. Cell, 166, 907–919. 10.1016/j.cell.2016.07.004 27499021 PMC5418658

[pld337-bib-0038] Tunc‐Ozdemir, M. , & Jones, A. M. (2017a). Ligand‐induced dynamics of heterotrimeric G protein‐coupled receptor‐like kinase complexes. PLoS ONE, 12, e0171854 10.1371/journal.pone.0171854 28187200 PMC5302818

[pld337-bib-0039] Tunc‐Ozdemir, M. , & Jones, A. M. (2017b). BRL3 and AtRGS1 cooperate to fine tune growth inhibition and ROS activation. PLoS ONE, 12, e0177400 10.1371/journal.pone.0177400 28545052 PMC5436702

[pld337-bib-0040] Ueguchi‐Tanaka, M. , Fujisawa, Y. , Kobayashi, M. , Ashikari, M. , Iwasaki, Y. , Kitano, H. , & Matsuoka, M. (2000). Rice dwarf mutant d1, which is defective in the alpha subunit of the heterotrimeric G protein, affects gibberellin signal transduction. Proceedings of the National Academy of Sciences USA, 97, 11638–11643. 10.1073/pnas.97.21.11638 PMC17253 11027362

[pld337-bib-0041] Ullah, H. , Chen, J. G. , Young, J. C. , Im, K. H. , Sussman, M. R. , & Jones, A. M. (2001). Modulation of cell proliferation by heterotrimeric G protein in Arabidopsis. Science, 292, 2066–2069. 10.1126/science.1059040 11408654

[pld337-bib-0042] Urano, D. , & Jones, A. M. (2014). Heterotrimeric G protein‐coupled signaling in plants. Annual Review of Plant Biology, 65, 365–384. 10.1146/annurev-arplant-050213-040133 PMC4861148 24313842

[pld337-bib-0043] Urano, D. , Jones, J. C. , Wang, H. , Matthews, M. , Bradford, W. , Bennetzen, J. L. , & Jones, A. M. (2012). G protein activation without a GEF in the plant kingdom. PLoS Genetics, 8, e1002756 10.1371/journal.pgen.1002756 22761582 PMC3386157

[pld337-bib-0044] Urano, D. , Miura, K. , Wu, Q. , Iwasaki, Y. , Jackson, D. , & Jones, A. M. (2016). Plant Morphology of Heterotrimeric G Protein Mutants. Plant and Cell Physiology, 57, 437–445. 10.1093/pcp/pcw002 26755691 PMC4900173

[pld337-bib-0045] Urano, D. , Phan, N. , Jones, J. C. , Yang, J. , Huang, J. , Grigston, J. , … Jones, A. M. (2012). Endocytosis of the seven‐transmembrane RGS1 protein activates G‐protein‐coupled signalling in Arabidopsis. Nature Cell Biology, 14, 1079–1088. 10.1038/ncb2568 22940907 PMC3463750

[pld337-bib-0046] Wang, L. , & Ruan, Y.‐L. (2013). Regulation of cell division and expansion by sugar and auxin signaling. Frontiers in Plant Science, 4, 163. 23755057 10.3389/fpls.2013.00163 PMC3667240

[pld337-bib-0047] Wetmore, R. H. , & Rier, J. P. (1963). Experimental induction of vascular tissues in callus of angiosperms. American Journal of Botany, 50, 418–430. 10.2307/2440311

[pld337-bib-0048] Yan, Q. , Wang, J. , Fu, Z. Q. , & Chen, W. (2017). Endocytosis of AtRGS1 Is regulated by the autophagy pathway after d‐glucose stimulation. Frontiers in Plant Science, 8, 1229 10.3389/fpls.2017.01229 28747924 PMC5506085

